# Investigating New Zealand radiation therapy student perceptions about their degree curriculum

**DOI:** 10.1186/s12909-022-03973-9

**Published:** 2022-12-23

**Authors:** Paul Kane, Tehmina Gladman, Sarah Stein, Julie A. Timmermans

**Affiliations:** 1 Department of Radiation Therapy, University of Otago Wellington, 23A Mein Street, Newtown, Wellington, 6242 New Zealand; 2 Medical Education Unit, University of Otago Wellington, 23A Mein Street, Newtown, Wellington, 6242 New Zealand; 3 Distance Learning, University of Otago, 145 Union Street, Dunedin, 9016 New Zealand; 4 Higher Education Development Centre, University of Otago, 362 Leith Street, Dunedin North, Dunedin, 9016 New Zealand

**Keywords:** Education, Curriculum, Radiation therapist, Health professional, Grounded theory, Student experience

## Abstract

**Background:**

Radiation Therapists (RTs) are a key professional grouping in the delivery of health services for cancer patients. The education of RTs in New Zealand has evolved in response to regulatory and clinical workforce requirements. To date, it has lacked a fundamental underpinning of educational theory. Stakeholders, including students, were canvassed for their perspectives on the drivers behind the current curriculum with a view to developing theory which could shape future curricular development.

**Methods:**

A focus group was conducted with eight student RTs enrolled at the time of the study. A process driven by Constructivist Grounded Theory principles was adopted for the analysis of the resulting data.

**Results:**

Four themes were established to represent the data: “Being” is prized over “doing”, Change is inevitable, A framework for Professional Identity formation and Modelling is key to learning.

**Conclusions:**

There is utility in exploring the student perspective around curriculum. The data suggest that students on this programme are engaged with the process of preparing for practice and the connected learning experiences. There is a focus on the patient and the personal values and qualities which result from that focus. While specialist knowledge and technical skills are required for delivering patient care, it is fully expected those aspects of the clinical role will significantly change over time. Even at this early stage in their careers, students recognise the development and need for professional identity formation. Role models are perceived to be a vital part of student learning, be they positive or negative. Scrutiny of the study findings provides reason to question some assumptions which are sometimes made about student practitioners based on factors such as age and gender and the assumed universal ability of practitioners to teach the next generation. The perspectives gained inform the next stage of data collection from this group and theory building that will be reported outside the confines of this article.

## Background

Radiation therapy is a key treatment modality in the treatment of cancer [1]. Treatment is usually delivered by health professionals whose title and training depends on jurisdiction [2, 3]. In New Zealand (NZ), the role is performed by Radiation Therapists (RTs). The Bachelor of Radiation Therapy (BRT), offered by the University of Otago Wellington, is the sole qualification leading to registration to practice as an RT in NZ. The BRT is a three-year ordinary degree which includes a two-week clinical placement in the first year and an 18-week placement in each of the second and third years. Academic investigation of the BRT curriculum has been sparse and has focused on very specific topics of interest [4–6]. This pattern reflects curriculum investigation of equivalent courses of study internationally [7–12]. While well established and producing competent practitioners, the BRT has no overarching theoretical underpinning for the design of its curriculum, unlike examples found in nursing, medicine, physiotherapy and occupational therapy [13–16]. That is not to say there is no sense of how knowledge is organised or made available to students as any curriculum design will require [17]; rather the BRT has evolved more as a response to changing clinical needs and the legal framework regulating NZ RTs than by taking a particular theoretical position. This evolution is mirrored by efforts in Europe to produce standardised learning outcomes, facilitating the comparison of national qualifications from EU member states. The main purpose of that work is to promote ready movement of labour [18]. What has not been commonly published are curriculum design processes like that reported by Buckley et al. which adopted a specific set of theoretical principles from the inception of the design process. Even this outlier project relates to a specialty course rather than a comprehensive pre-registration educational programme [19].

**Fig. 1:**
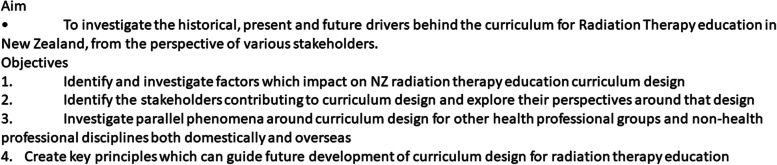
Aim and Objectives of overarching project this article contributes to

A large-scale project was initiated, Fig. 1 details the aim and objectives of that project. The stakeholders were identified as including radiation therapists, educators, clinical tutors, managers and students amongst others. Giving students a voice is well established as a regular and useful part of educational research; helping inform an appreciation of quality [20] contributing to their individual learning [21] and satisfaction in their role [22]. It also made sense to canvass students as they had a contemporary perspective of BRT learning experiences. Objective 2 of the larger project seeks perspectives from all stakeholders. This article presents an analysis of the student perspective, informing further data collection and exploring some of the ideas which will contribute to achieving the remaining objectives.

## Methods

### Overview

The authors hold with the constructivist view that an understanding of the world is a product of experience, discourse and socialisation. As the purpose of the overarching project was to build theory where none existed it seemed appropriate to take an approach driven by Constructivist Grounded Theory (CGT) principles [23]. CGT has the ability to take data and build explanatory theory regarding the phenomena under investigation. The resulting theory or theories may sometimes be used to make predictions outside of the original context of the data.

### Participants

All students not on clinical placement at the time of data collection (first half of 2021) were invited by email, sent by Author 1, to take part in a focus group. Participants did not have to pre-register for the focus group, those who participated provided written informed consent.

Ethical approval for the study was provided by the University of Otago Human Ethics Committee (Approval Number 19/098).

### Process

A focus group was selected as it is a well established data collection tool which enables the capture of multiple perspectives simultaneously, the opportunity for peers to stimulate thought and encourage debate which mitigates some of the need for researchers to interpret [24–26]. At the time of the study, author 1 was on Research and Study Leave and was therefore unknown to year 1 participants and not involved in teaching of the year 3 participants. The focus group was facilitated by author 2 who is familiar with the BRT but does *not* teach on it and is *not* generally known to the student body. It was run on a face-to-face basis to promote interaction between participants. No video option was offered to students off-campus as they were on placement, which would have presented scheduling and logistical difficulties. A question schedule (Table 1), used across all aspects of the larger study, explored participant views on what it means to be an RT, the influences affecting those views and what (if any) other factors, groups or individuals had an influence on what it means to be an RT. The focus group occurred in May 2021. Discussion was audio recorded and transcribed verbatim by an independent transcriber.

**Table 1:** Interview Schedule. The project is investigating curriculum. A working definition of curriculum is “The total set of experiences that a student or learner will encounter in the learning process”. The purpose of that curriculum is to prepare students for practice as Radiation Therapists. With those two ideas in mind I have some questions for you to consider

Initial Question	Prompts or Follow Up Questions	Rationale	Notes
What does it mean to be a radiation therapist?	A personal perspective is appropriate Has your understanding of that changed since you became a student RT? What experiences on the programme have shaped your perspective? What do these terms mean to you: competent, qualified, graduate, practitioner Do you think all student RTs would think what you think? Why do you say that?	Establish participant’s perspective	
Who or what determines what it means to be a radiation therapist?	Can you identify the groups or factors that influence or drive what it means to be a radiation therapist? Some more than others? To what degree? Why has that been the case? Will those factors or parties determine those things in the future? Are there “things” which make a person a radiation therapist? Such as: Values, behaviours, habits, practices, specialist knowledge? Where and how are those “things” established? Is there a point someone “becomes” an RT? How do they get to that point? Who shapes that? Curriculum – what does it mean do you? (lead to next main question)	Can the participant explain their perspective?	
What makes you say those are the things which determine what is means to be a radiation therapist?	Tell me about key experiences as a student that influence your answer? Has your perspective changed as you progressed through the programme? - academic or clinical experiences, experiences from different clinical centres	Influences on the participant – allow me to see through their lens	
What is your opinion about those factors determining what it means to be a radiation therapist?	Is the status quo, as you understand it, appropriate? Would you like to see any of this change? Can you elaborate? Will “being a RT” remain the same now and in the future? Can you elaborate? In what ways do you think the COVD-19 pandemic has affected things? Is that likely to remain the case?	Has the participant a view of the future, what basis does that have?	
Is there anything we have not addressed today that you would like to mention or comment on?		Allow for burning issues and door handle moments	

### Analysis

The transcript was imported into nVivo 11 (R) for analysis. Data were sorted by Author 1 using a two-stage coding process. Broader line by line initial codes were followed by a second more focused set of codes designed to establish links between ideas in the initial codes. Memos were used to assist in constructing themes to explain the meaning of the data [23]. Author 2 independently performed a coding pass. Discussion of coding and resulting themes resolved any points of contention and achieved consensus. The analysis sought insight on student perspectives of the role they were preparing for and what learning experiences were useful to them.

### Reflexive statement

The purpose of this study was to contribute to a wider examination of why RTs are taught the way they are in NZ and to construct theoretical principles which would guide future developments of the BRT curriculum. This is a topic which has not been addressed previously by formal investigation. Author 1 is an academic from an overseas clinical radiation therapy background. He was not part of the team who devised the BRT but he has taught on the programme for over a decade and has experience of both qualitative and educational research. Author 2 is part of the same institution but comes from a non-health, non-clinical academic background, her role is one of academic development support for a section of the University which is dominated by clinically based teaching staff. Her research experience extends across the quantitative and qualitative paradigms and is focused on building the evidence base for good teaching and learning practice. Authors 3 and 4 serve as doctoral candidate supervisors for author 1, both have roles with an educational research focus.

### Quality

Charmaz proposes four criteria to consider in her approach to establishing quality. These are credibility, originality, resonance and usefulness [23, 27].

#### Credibility

This specific study is a component part of a much larger study design which collects data from a complete set of identified stakeholders. The analysis of the data is informed by a well documented set of principles and is performed by researchers with the requisite skills and experience.

#### Originality

The study is novel in that no comparable work has been performed in this context before, seeks to provide data driven evidence to inform future development and practice and build theory where none currently exists.

#### Resonance

This study aims to give voice to stakeholders and represent what they wish to communicate to those who control their learning experience. The data collection approach permitted participants to determine the specifics and tone of the data collected.

#### Usefulness

While not completed in this particular study, it does form an integral part of a much larger investigation which will inform decisions around the educational experience of an important group of health professionals.

## Results

### Participant details

Eight students took part in a focus group lasting 1 hour. Three from year 1 and five from year 3 of the programme. Six identified as female, two as male. Ages ranged from 18 to 26 with a mean of 22. Six identified as NZ European, one as Chinese and one as African. These demographic markers are highly representative of the total student population, which is predominantly female, under 25 years of age and of New Zealand European ethnicity.

### Themes

Analysis produced four overarching themes;” Being is prized over “Doing”, Change is inevitable, a framework for professional identity formation and modelling is key to learning. Table 2 lays out the codes and their connection to the themes constructed. We discuss the themes in detail below, providing example supporting quotations from the transcript. The quotations used are representative of the entire group discussion.

**Table 2:** Linking Focused Codes to constructed themes

Theme	Related Codes
**Being is prized over doing**	Being an RT is about qualities and values Named values or attributes Taught versus intrinsic Influences shaping values RT role is unique
**Change is inevitable**	Technology is important, the role will change
**A framework for professional identity formation**	Certification Now I am an RT change of perception Teacher and practitioner
**Modelling – is key to learning**	Negative model Classroom does prepare Clinical experience shapes perception Theory practice gap what is curriculum

The column on the left indicates the final constructed themes, the column on the right indicates the codes which were linked to those final themes (also known as categories).

### “Being” is prized over “doing”

When asked what it meant “to be” a Radiation Therapist (RT), the conversation with participants focussed on the values, qualities and attributes that a radiation therapist possesses (what an RT *is*) rather than academic achievement, technical skills or knowledge (what an RT *does*). The patient centric nature of that “being” resonated strongly with participants.

*“when I got into this degree, I didn’t realise how heavily patient focused it was. I was very much… thinking it was more about the technology and the treating of cancer…. but it’s definitely so strongly focused on the patient which I love”*

There was recognition of the knowledge and skills required to provide a specialised form of treatment, but the relationships formed with patients appeared more highly prized including advocating for, supporting, and identifying patients’ holistic needs. Technology, while not without its “wow” factor is a means to an end, allowing RTs to provide care for their patients.

*“unless you had an interest in helping people and treating people...I don’t think you’re going to hang around long enough to be able to put up with maybe the more challenging aspects of dealing with someone who’s upset.”*

Ultimately, if it were not the knowledge and technology currently used, it would be another version of it. The person behind the role will have the same motivations and personal traits.

### Change is inevitable

This theme centred on how the technical knowledge an RT requires is perceived. That knowledge feeds into a set of practical skills across clinical practice. The combination of knowledge and practical tasks determines *what* RTs do as part of their everyday practice. It was acknowledged that, as research expands the RT knowledge base and as new technology becomes available, the “what” will change, and that change is actively pursued.

*“I guess that kind of speaks to...how different it was...compared to how it is now, so I feel like that’s probably something that will continue to happen as we go forward...hopefully always...empathic and patient centred”*

The contrast highlighted by participants, is the desire that *how* RTs work should not change with respect to applying professional judgement and working towards a good outcome for patients. The ability to take current knowledge and technology and use both appropriately is a consistent feature of practice. For example, those who have been in practice for 10 years do things differently to when they first entered practice. These student participants fully expect to know and use different tools 10 years from now. Their approach to how that work is done is likely to be consistent with what and how they learn now. A specific example provided was greater levels of automation which potentially freed them to spend more time with patients. Additionally, participants expressed a strong desire for the curricular experience to prepare them for change and evolution, which they hold to be inevitable and, in many cases, desirable.

*“I’d hope that only the technology would change but ...we [will] still treat the patient and...all that side of it would not change”*

### A framework for professional identity formation

This theme was constructed to reflect participant awareness of professional identity formation taking place. There was a strong sense that a key factor impacting professional identity formation related to self-perception, if a student does not feel they have made the transition to becoming a professional, then it has not happened.

“*Interviewer

*So what do you think makes you feel that you’re not quite there yet? Is there anything that they do or is it something that’s inside you that feels that way?*

*Participant

*Um, I think it’s probably me”*

Another key element concerned how individuals were regarded by others, often those the students were supervised by as they were learning. Participants anticipated distinct, future events marking their transition to becoming professionals.

*“Cos[sic] they [clinical Radiation Therapists] do push you to do things and say, ... you’re just as competent as we are”*

Professional identity does not simply encompass what is known, or what can be done but also how participants think and respond to their environment. The BRT learning experience appears to provide a framework which allows students to recognise when they have moved from being students to practitioners.

*“Up to that, up to that point, I can know the things but I’m not one”*

### Modelling is key to learning

This theme addresses a range of ideas relating to behavioural and learning models which students are exposed to and their impact on learning experience. Behavioural and professional practice models can be positive, providing inspiration; or negative, establishing patterns of behaviour or practice to avoid.

*“I think there are, you can see the staff members as you can see them as like good radiation therapists and maybe one that’s not quite, not someone that you would want as a radiation therapist.”*

Not everyone students interacted with appeared interested in student learning.

*“Cos [*sic*] they’re [clinical Radiation Therapists]...highly variable as to...how...keen they are on having students involved and stuff so... some don’t get you involved as much and then some are...really interested and wanting you to learn“.*

Participants discussed actively seeking models from the beginning of their learning experience.

*“...the staff...you watch them when you’re on a placement and you see how skilled they are and how much they know and you’re like, okay, I need to be like that.”*

There was little distinction drawn between the perceived value of models demonstrated in the classroom or clinical context.

*“the fact that...our lecturers are radiation therapists and that influences us, I think is a good thing because they...keep it real with us and we know exactly what we’re getting into”*

This theme also includes the influence of models on the learning experience quality. Value was placed on learning experiences which were more than simple knowledge transfer, instead bringing out the best from students, drawing on prior knowledge, experience and existing qualities.

*“So they kind of teach us to understand ourselves so we can understand the patients”*

## Discussion

As indicated previously, this paper reflects one set of data from a much larger study. Outside the context of the complete dataset, theory building is not practical. The discussion of the findings detailed above highlights some of the ways our data aligns with current literature and additionally ways in which some misconceptions, which unfortunately prevail in the discussions between stakeholders, can be challenged. We also indicate the next steps in the use of our analysis.

### Misconception 1: of course they said that, they are young women!

Entry to the BRT is competitive and radiation therapy is a niche specialty in the local healthcare system, fewer than 400 practitioners are registered in New Zealand [28]. It is interesting that aspects of being a practitioner, such as values and patient-centred behaviors were prioritised over specialised knowledge, which arguably is what makes the professional grouping niche. This emphasis on patient-centred values and the resulting care provided is not unique to radiation therapy and is to be found amongst other health professional groupings [29, 30]. Technology applied for a very specific purpose is central to the practice of a Radiation Therapist and participants readily acknowledged the need for competent use of this technology in their future practice. It is noteworthy that the technology is viewed as merely a means to an end by participants, with high quality patient care being that end. Our focus group participants primarily identified as female (which coincidentally aligns with the makeup of both the entire student body enrolled at the time and the professional grouping in the NZ context and abroad). There has been a longstanding idea that women gravitate towards more people orientated roles, characterized as “women are interested in people, men are interested in things” [31]. However, recent literature has challenged these assertions as being reductionist. For example, it seems the gendered make-up of any profession is not inevitable; offering learning experiences and perspectives which challenge societal gender norms can shift future career choices [32]. Another recent study seems to indicate that while women do seem to have stronger preferences compared to men when it comes to prioritizing “people” over “brains” or “brawn”, men and women do not necessarily fit into opposite ends of the spectrum for role preference [33]. While our data are insufficient to lay the gender stereotype to rest it does seem to confirm that those entering the RT profession are primarily interested in people.

### Misconception 2: digital natives handle change better?

Whilst still in the pre-registration environment, these participants fully anticipate the nature of their specialised knowledge and skill set will change over the course of their career. A commonly held misconception – that of the “digital native” [34] – is that younger generations accept technology and appreciate the advantages and fluid nature of technological development; however, examination of the literature suggests otherwise [35, 36]. The field of Radiation Therapy along with much of the healthcare system has often adopted new and improved technologies to improve efficiency or in the interests of improved patient care, for example, the first computer controlled Linear Accelerators (the key piece of equipment used by RTs) appeared in the 1970s [37]. As such, this attitude does not make current students remarkable. Instead, we would suggest that this feature of participant discussion is more indicative of the type of learning experiences that students have been exposed to, ones which steer them towards anticipation of ongoing change in the technological tools used in professional practice.

### Misconception 3: young people don’t know who they are!

The focus group data demonstrated evidence of professional identity formation, which is interesting given students are in the pre-professional practice environment. Our group represents the age demographic of the student body very well. Entry to the programme is possible directly from High School, making the youngest students who enroll 17 years old. The focus group participants are arguably still in the formative stages of their personal identity formation which will likely have some impact on professional identify development. Kegan’s framework for identify formation has utility here, as the structure placed around the different years of study in the BRT programme place students in progressively more complex scenarios, providing opportunities to construct and situate themselves in more complex systems of understanding the world [38]. Professional identity does need to be separated from professionalism or professional value sets. Rather than values leading to observable and therefore measurable behaviours, professional identity is more closely related to how the practitioner perceives themselves [22, 39]. So regardless of what they know, are students in fact thinking and feeling like radiation therapists? Participants reported that to be considered professionals it was necessary they have their own sense of having made that transition. It seems noteworthy that students have some sense of professional identity development which reflects well on the BRT, especially as there is evidence that curriculum design for health practitioners should not only establish competence but also allow for professional identity formation [40, 41].

### Misconception 4: anyone can teach!

The theme concerning role modelling has been an interesting one to consider. Participants report a clearly held identification of the patient as their focus. That approach appears to have been modelled by their teachers, both academic and clinical, and the practitioners who supervise them on placement. The work of Brown and Collins laid out a variety of teaching strategies in their Cognitive Apprenticeship theory of learning with modelling generally placed at the top [42, 43]. Modelling is an opportunity for experts in a field to demonstrate aspects of a role in such a way that students can construct a conceptual framework, which is what participants in this study appear to be identifying. The use of this theory has been well established in medical and nursing education for some time and there is an extensive literature which confirms its utility from both a teacher and learner perspective [44–48]. Additionally, Stalmeijer et al. indicated that student participants in their study saw teachers with less well-developed teaching skills as problematic. Participants in our study reported negative role models and interactions with practitioners where a desire to see students learn was not evident [45]. This appears to conflict with the apparent focus on patients. Ensuring the growth of the well-rounded practitioners of tomorrow would seem to be a useful way to contribute to quality patient care.

The analysis of this data has provided insight into what student RTs value in terms of the preparation they receive for professional practice. The focus group had eight participants from a total enrolled student body of 75 individuals. Using our analysis, we developed a survey tool to administer to the entire population of student RTs to establish if our analysis resonated with them. We will report on that phase separately.

## Limitations

This study is arguably limited by the fact it only canvassed the students enrolled on the BRT at the time of the study. The focus group data were also collected at a single time point and students are enrolled for 3 years on the degree. The authors recognise there would be value in pursuing these questions in a more longitudinal manner, mapping any trends or deviations as different student cohorts enrol and progress through the BRT programme. The recruitment timeframe was when year two students were on placement right across New Zealand, excluding them from participation in the face-to-face focus group. Their input was canvassed in the online survey developed from the focus group data which is reported separately from this article. A grounded theory analysis usually means the reader expects a statement on saturation. Charmaz recommends seeking “saturation of categories” and to do so by gathering additional data.(21 page 113) This study had limitations in terms of time and resource available to do so. Therefore, while the themes constructed are supported by the data, we are reluctant to make the claim that saturation has been reached. The reader should also consider this article reports on only a small part of the data collected to address the overarching project objectives. Saturation across the project is something we will have more confidence in claiming.

## Conclusion

Exploration of student perspectives when examining the drivers of curriculum design provides important insights concerning the learning experiences students have. Data from this study suggest students appear to be engaged with the process of preparing for practice and recognise learning opportunities and experiences which will be useful to them in that process. Participants have reported a strong focus on the patient, an awareness that the tools of their practice are subject to change, opportunities to develop a professional identity and an appreciation of the value of role models for their learning. A closer look at those perspectives and some relevant literature gives us a basis to challenge some misconceptions. The values held by students, their ability to handle changing technology, the development of their professional identity and recognition of positive role models have less to do with their age or gender and more to do with an appropriate set of learning experiences.

## Data Availability

The dataset used and analysed during the current study is available from the corresponding author on reasonable request.

## Abbreviations

BRT: Bachelor of Radiation Therapy
RT: Radiation Therapist
CGT: Constructivist Grounded Theory
